# Public Awareness, Individual Prevention Practice, and Psychological Effect at the Beginning of the COVID-19 Outbreak in China

**DOI:** 10.2188/jea.JE20200148

**Published:** 2020-10-05

**Authors:** Bingfeng Han, Tianshuo Zhao, Bei Liu, Hanyu Liu, Hui Zheng, Yongmei Wan, Jiayi Qiu, Hui Zhuang, Fuqiang Cui

**Affiliations:** 1 Department of Epidemiology and Biostatistics, School of Public Health, Peking University, Beijing, China; 2 National Immunization Program, Chinese Center for Disease Control and Prevention, Beijing, China; 3 Department of Laboratorial Science and Technology, School of Public Health, Peking University, Beijing, China; 4 Department of Microbiology and Center of Infectious Diseases, School of Basic Medical Sciences, Peking University, Beijing, China

**Keywords:** COVID-19, awareness, prevention practice, psychology

## Abstract

**Background:**

The COVID-19 has spread to more than 200 countries and territories. But less is known about the knowledge, protection behavior and anxiety regarding the outbreak among the general population.

**Methods:**

A cross-sectional, population-based online survey was conducted in China and abroad from January 28 to February 1, 2020. Socio-demographic information was collected and knowledge scores, practice scores, anxiety scores and perceived risk were calculated. General linear model and binary logistic regression were used to identify possible associations.

**Results:**

We included 9,764 individuals in this study, and 156 (1.6%) were from Hubei Province. The average knowledge score was 4.7 (standard deviation, 1.0) (scored on a 6-point scale); 96.1% maintained hand hygiene, and 90.3% of participants had varying levels of anxiety. People in Hubei Province were the most anxious, followed by those in Beijing and Shanghai. People who had experienced risk behaviors did not pay more attention to wearing masks and hand hygiene.

**Conclusions:**

The public had high awareness on knowledge of COVID-19 outbreak, and a high proportion of people practiced good hand hygiene behavior. Many people claimed anxiety, especially in heavily affected areas during pandemic, suggesting the importance of closing the gap between risk awareness and good practice and conduct psychological counseling to public and patients.

## INTRODUCTION

In December 2019, a cluster of patients with pneumonia of unknown cause (named COVID-19 on February 12, 2020^1^) was linked to a seafood wholesale market in Wuhan, Hubei, China.^2^^,^^3^ In the next 2 months, the outbreak spread to the rest of the provinces. On January 8, 2020, a novel coronavirus (named SARS-CoV-2 a month later^4^) was laboratory confirmed as the cause of the outbreak, similar to the severe acute respiratory syndrome coronavirus (SARS-CoV)^5^ that caused severe acute respiratory syndrome outbreaks in China in 2003 and the Middle East respiratory syndrome coronavirus (MERS-CoV)^6^ that caused severe respiratory disease outbreaks in the Middle East in 2012. Since January 3, China began to report the outbreak information to the World Health Organization (WHO) regularly.^7^ The test kit was initially developed on January 10. As of January 28 (when this study started), COVID-19 infection caused 5,974 cases in Mainland China and the number was growing dramatically.^8^ As of February 1 (when this study ended), 14,380 cases were reported.^9^ Currently COVID-19 has caused a global pandemic, with more than 6 million cases and more than 370,000 deaths reported in 216 countries and territories by the end of May 2020^10^ (See eFigure 1 for detailed timeline).

The dissemination of epidemic information and personal protection knowledge plays an important role in preventing and controlling the outbreak. The evidence suggested that human-to-human transmission had occurred among close contacts,^11^^,^^12^ and the basic reproductive number (R_0_) was estimated approximately as 2.68.^13^ The outbreak coincided with the Chinese Lunar New Year, and the mobility of people visiting relatives and friends increased, which also increased the difficulty of prevention and control. To improve the awareness of self-protection among the general population, the National Health Commission had released six versions of the new coronavirus pneumonia prevention and control protocol^14^ and published guidelines for public prevention, recommending minimizing outings, wearing a mask, and keeping hands clean.^15^ These suggestions, as well as daily outbreak information, are transmitted to the public through television, mobile phones, the internet, and other means. People have discussed the outbreak and expressed their views in “we media” era during the spread of the outbreak. Due to the rapid spread of the outbreak and the control activities, some people developed anxiety and fear. Guidance on emergency psychological crisis intervention was published for patients, medical professionals, and the general public to prevent the psychological damage caused by the outbreak and promote social stability.^16^ Psychological assistance hotlines have been set up in all cities and counties, providing free 24-hour service.^17^

To our knowledge, there has been no article reporting the current public awareness and psychological status of Chinese people focused on virus and epidemiology of outbreak. Understanding the public knowledge, practice of prevention, and psychological status can improve effectiveness of health risk communications, and analyzing their demographic differences can help avoid unequal protection across society.^18^ Therefore, at the beginning of the COVID-19 outbreak in China, we designed an online questionnaire to measure the knowledge and practice, providing references for reassuring citizens and outbreak control.

## METHODS

### Study population

A cross-sectional, population-based online survey was conducted from January 28 to February 1, 2020. It was an open online questionnaire for all the population aged 18 years or above residing in China and abroad. Those willing to respond could complete the questionnaire by mobile phone or by computer.

### Online questionnaire

We designed a structured Chinese questionnaire and collected data on Wenjuanxing, an online platform providing functions equivalent to Amazon Mechanical Turk. In the questionnaire, we collected the following information: (1) the socio-demographic information of the respondents; (2) knowledge of COVID-19, including the transmission routes, susceptible population, incubation period and response principle (total, 6 questions); (3) practices of preventive measures against COVID-19, including wearing masks, personal hygiene practices, avoidance of contact with high risk group (total, 4 questions); (4) anxiety towards COVID-19, using the five-question short form of the State-Trait Anxiety Inventory (STAI) to measure anxiety; (5) risky behavior, defined as coming in contact with someone with a confirmed or suspected case; and (6) perceived risks, including five categorical options (No risk, Low risk, Medium risk, High risk, Very high risk). The questionnaire consisted of 33 questions and can be completed in 3–5 minutes (see eMaterials 1).

### Data management and statistical analysis

We used SPSS (version 20.0, IBM, Armonk, NY, USA) and STATA (version 15.1, StataCorp LLC, College Station, TX, USA) for data cleaning and statistical analysis. Categorical variables were expressed as absolute and relative frequencies in different groups. According to the following principle, we calculated scores: (1) Knowledge of COVID-19, measured using “knowledge scores”: questions were scored “1 point” (correct answer) or “0 point” (wrong answer or incomplete answer in multiple choices); (2) Practices of preventive measures against COVID-19, measured using “practices scores”: questions were scored “1 point” (correct practice) or “0 point” (wrong practice); (3) Anxiety towards COVID-19, measured using a five-point Likert-type scale to ascertain the degree of anxiety for five questions (from 1 to 5, 1 = never, 2 = little, 3 = sometimes, 4 = often, 5 = always; total “anxiety scores” ranged from 5 to 25; in order to compare the relative level of anxiety among people with different characteristics, we defined anxiety scores of ≤25th percentile as low anxiety levels, 25th–75th percentile as moderate anxiety levels, and ≥75th percentile as high anxiety levels); (4) risk behavior, categorized as a binary variable (1 = have, 0 = don’t have); and (5) perceived risks, which were divided into five order categories according to severity (from 1 to 5, 1 = no risk, 2 = low risk, 3 = medium risk, 4 = high risk, 5 = very high risk).

Direct standardized questionnaire was used to measure knowledge scores, practices scores, and anxiety scores on different ages and educations of population to improve comparability among provinces. General linear modeling (GLM) was used to identify associations of sociodemographic factors (eg, sex, age, and education) with knowledge scores, practices scores, and anxiety scores. We used trend linear regression to test the change trend of scores in people with different age and education. Binary logistic regression was used to explore the relationship between risk behaviors and wearing masks and hand hygiene, and odds ratios (ORs) and their 95% confidence intervals (CIs) were calculated. The component ratio was used to describe the channel through which people obtained information.

The spatial data analyses were conducted using ArcGIS (version 10.2, ESRI Corp, Redlands, CA, USA). The significance level was considered when *P* values were less than 0.05.

### Quality control

We monitored the progress of the survey every day. After the deadline, we checked the accuracy of data, and excluded the questionnaire if (1) the age range was below 18 or above 100 years, (2) the answering time was less than 150 seconds, or (3) there were logical contradictions between the answers to the questionnaire.

### Ethical approval

This study was approved as ethical exemption by the Peking University Health Science Center Ethics Committee (IRB00001052). All subjects participated in the survey voluntarily, and the information in the database was completely de-identified.

## RESULTS

### Study participants and characteristics

In total, 10,966 individuals participated in this online survey. Among these, 1,202 were excluded due to out of age range or incomplete questionnaire, and the rate of completeness was 89.0% (9,764/10,966) (See eFigure 2).

**Figure 1.**
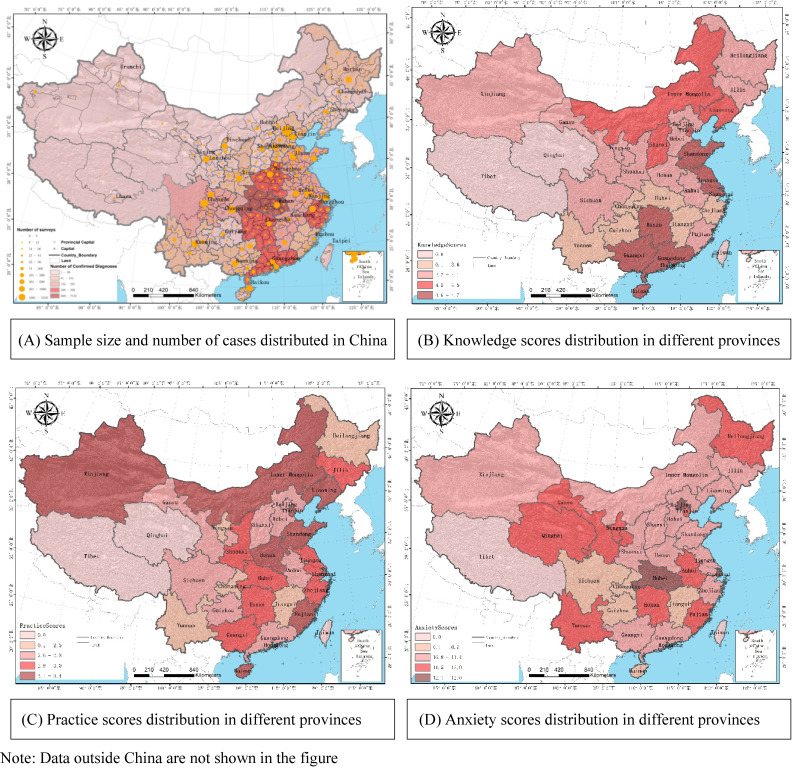
Distribution of case numbers, sample sizes, and knowledge/practice/anxiety scores by provinces in China

The participants of this study covered all 31 provincial administrative regions in Mainland China. Among the 9,764 eligible participants, 156 (1.6%) were from Hubei Province, 9,408 (96.4%) were from other provinces in China (Mainly from Beijing, Shandong, Sichuan, and Guangxi), and 200 (2.0%) were from abroad; 3,278 (33.6%) was male; average age was 38.0 (standard deviation [SD], 12.0) years; 9,281 (95.1%) were with senior high school education or above; 7,841 (80.3%) were urban people; and 6,681 (67.8%) were married (Figure 1A, Table 1).

**Table 1.** The characteristic of valid participants in online survey, China, 2020

Characteristics	From Hubei province *n* (%)	From other provinces in China *n* (%)	From abroad *n* (%)	Total *n* (%)
**Gender**				
Female	98 (62.8)	6,257 (66.5)	131 (65.5)	6,486 (66.4)
Male	58 (37.2)	3,151 (33.5)	69 (34.5)	3,278 (33.6)
**Age group, years**				
<30	56 (35.9)	2,611 (27.8)	58 (29.0)	2,725 (27.9)
30–39	44 (28.2)	2,741 (29.1)	73 (36.5)	2,858 (29.3)
40–49	35 (22.4)	2,309 (24.5)	38 (19.0)	2,382 (24.4)
≥50	21 (13.5)	1,747 (18.6)	31 (15.5)	1,799 (18.4)
**Education**				
Junior high school and below	5 (3.2)	474 (5.0)	4 (2.0)	483 (4.9)
Senior high school	20 (12.8)	1,279 (13.6)	16 (8.0)	1,315 (13.5)
Bachelor’s degree	91 (58.3)	5,372 (57.1)	86 (43.0)	5,549 (56.8)
Master’s degree or above	40 (25.6)	2,283 (24.3)	94 (47.0)	2,417 (24.8)
**Marriage**				
Married	91 (58.3)	6,392 (67.9)	135 (67.5)	6,618 (67.8)
Unmarried	62 (39.7)	2,664 (28.3)	63 (31.5)	2,789 (28.6)
Divorced	3 (1.9)	255 (2.7)	2 (1.0)	260 (2.7)
Widowed	0	56 (0.6)	0	56 (0.6)
Other	0	41 (0.4)	0	41 (0.4)
**Occupation**				
Medical professional	23 (14.7)	1,717 (18.3)	18 (9.0)	1,758 (18.0)
Labors	5 (3.2)	643 (6.8)	13 (6.5)	661 (6.8)
Teachers and researchers	32 (20.5)	1,855 (19.7)	45 (22.5)	1,932 (19.8)
C&S personnel	30 (19.2)	1,910 (20.3)	58 (29.0)	1,998 (20.5)
Students	37 (23.7)	1,161 (12.3)	33 (16.5)	1,231 (12.6)
Other	29 (18.6)	2,122 (22.6)	33 (16.5)	2,184 (22.4)
**Residence**				
Rural	44 (28.2)	1,849 (19.7)	30 (15.0)	1,923 (19.7)
Urban	112 (71.8)	7,559 (80.3)	170 (85.0)	7,841 (80.3)
**Total**	**156**	**9,408**	**200**	**9,764**

C&S, commercial and service personnel.

### Distribution of knowledge scores

Average knowledge score of all respondents was 4.7 (SD, 1.0). 77.3% of respondents (7,549/9,764) accurately knew the transmission route of COVID-19, and 95.4% (9,311/9,764) knew the incubation period of the disease.

Males had slightly lower knowledge scores than females (mean 4.6 vs 4.7; *P* < 0.05). People over 50 years had lower knowledge scores (mean, 4.5; *P* < 0.01). With the increase of education, people obtained higher knowledge scores (*P* < 0.01). Knowledge scores of medical personnel were higher than those in people with other occupations. There was no significant difference of knowledge scores in people with different marriage status (Table 2).

**Table 2.** Knowledge scores, practice scores and anxiety scores by subject characteristics in General Linear Model (GLM), China, 2020

Characteristics	Number	Knowledge scores			Practice scores		

		Mean	β	t	Mean	β	t
**Gender**							
Female^Ref^	6,486	4.7			3.2		
Male	3,278	4.6	0.0	−2.3^a^	2.9	−0.2	−11.9^b^
**Age group, years**							
<30^Ref^	2,725	4.7			3.3		
30–39	2,858	4.8	0.1	2.6^a^	3.2	0.0	−1.3
40–49	2,382	4.7	0.0	1.0	3.0	−0.2	−6.5^b^
≥50	1,799	4.5	−0.1	−3.0^b^	2.8	−0.4	−10.9^b^
**Education**							
Junior high school and below^Ref^	483	4.0			2.8		
Senior high school	1,315	4.3	0.4	7.2^b^	3.0	0.2	3.3^b^
Bachelor’s degree	5,549	4.8	0.7	14.7^b^	3.1	0.2	5.7^b^
Master’s degree or above	2,417	4.9	0.9	15.9^b^	3.2	0.3	5.4^b^
**Marriage**							
Married^Ref^	6,618	4.7			3.1		
Unmarried	2,789	4.7	0.0	−0.6	3.2	0.0	0.4
Divorced	260	4.6	0.0	−0.1	3.1	0.0	0.3
Widowed	56	4.4	−0.1	−0.8	2.8	−0.2	−1.5
Other	41	4.3	−0.3	−2.0^a^	3.0	0.0	−0.1
**Occupation**							
Medical professional^Ref^	1,758	4.9			3.2		
Labors	661	4.3	−0.3	−5.7^b^	2.8	−0.2	−4.4^b^
Teachers and researchers	1,932	4.8	−0.2	−6.4^b^	3.1	−0.1	−2.1^a^
C&S personnel	1,998	4.7	−0.2	−7.0^b^	3.1	−0.1	−3.1^b^
Students	1,231	4.7	−0.2	−4.7^b^	3.2	−0.1	−3.4^b^
Other	2,184	4.6	−0.3	−7.8^b^	3.0	−0.1	−3.9^b^
**Urban/Rural**							
Rural^Ref^	1,923	4.6			3.1		
Urban	7,841	4.7	0.0	1.0	3.1	0.1	3.2^b^
**Area**							
Hubei province^Ref^	156	4.5			3.3		
Other provinces in China	9,408	4.7	0.2	3.0^b^	3.1	−0.1	−1.8
Abroad	200	4.6	0.0	0.4	2.9	−0.4	−4.5^b^
**Total**	9,764	4.7			3.1		

Characteristics	Number	Anxiety scores			

		Low	Moderate	High	χ^2^
**Gender**					
Female	6,486	1,654 (25.5)	2,845 (43.9)	1,987 (30.6)	156.0^b^
Male	3,278	1,178 (35.9)	1,412 (43.1)	688 (21.0)	
**Age group, years**					
<30	2,725	633 (23.2)	1,239 (45.5)	853 (31.3)	266.8^b^
30–39	2,858	711 (24.9)	1,221 (42.7)	926 (32.4)	
40–49	2,382	755 (31.7)	1,027 (43.1)	600 (25.2)	
≥50	1,799	733 (40.7)	770 (42.8)	296 (16.5)	
**Education**					
Junior high school and below	483	198 (41.0)	211 (43.7)	74 (15.3)	141.4^b^
Senior high school	1,315	497 (37.8)	546 (41.5)	272 (20.7)	
Bachelor’s degree	5,549	1,538 (27.7)	2,440 (44.0)	1,571 (28.3)	
Master’s degree or above	2,417	599 (24.8)	1,060 (43.9)	758 (31.4)	
**Marriage**					
Married	6,618	1,987 (30.0)	2,865 (43.3)	1,766 (26.7)	42.4^b^
Unmarried	2,789	709 (25.4)	1,254 (45.0)	826 (29.6)	
Divorced	260	101 (38.8)	94 (36.2)	65 (25.0)	
Widowed	56	20 (35.7)	29 (51.8)	7 (12.5)	
Other	41	15 (36.6)	15 (36.6)	11 (26.8)	
**Occupation**					
Medical professional	1,758	461 (26.2)	735 (41.8)	562 (32.0)	103.0^b^
Labors	661	260 (39.3)	263 (39.8)	138 (20.9)	
Teachers and researchers	1,932	602 (31.2)	872 (45.1)	458 (23.7)	
C&S personnel	1,998	579 (29.0)	879 (44.0)	540 (27.0)	
Students	1,231	270 (21.9)	573 (46.5)	388 (31.5)	
Other	2,184	660 (30.2)	935 (42.8)	589 (27.0)	
**Urban/Rural**					
Rural	1,923	582 (30.3)	871 (45.3)	470 (24.4)	10.5^b^
Urban	7,841	2,250 (28.7)	3,386 (43.2)	2,205 (28.1)	
**Area**					
Hubei province	156	26 (16.7)	72 (46.2)	58 (37.2)	15.2^b^
Other provinces in China	9,408	2,749 (29.2)	4,103 (43.6)	2,556 (27.2)	
Abroad	200	57 (28.5)	82 (41.0)	61 (30.5)	
**Total**	**9,764**	**2,832**	**4,257**	**2,675**	

C&S, commercial and service personnel; Ref, reference group.

^a^*P* < 0.05.

^b^*P* < 0.01.

After adjustment for age and education, we found differences existing among provinces: Guangxi, Guangdong, Hainan, and other southern provinces of China had higher knowledge scores (Figure 1B). The knowledge score from Hubei participants was significantly lower than other provinces participants’ score (β = 0.2, *P* < 0.01) but not statistically different with those in people abroad (β = 0.0, *P* = 0.72) (Table 2).

### Distribution of practice scores

The average practice score was 3.1 (SD, 0.9). 66.9% of respondents (6,535/9,764) were able to wear appropriate masks to prevent COVID-19 infection, and 81.5% (5,323/6,535) were able to change their masks regularly. 96.1% of people (9,386/9,764) maintained hand hygiene and washed their hands in time.

Males had lower practice scores than females (mean, 2.9 vs 3.2, *P* < 0.01). People over 40 years old had lower practice scores (*P* < 0.01). People with senior high school education and above and medical professionals obtained higher practice scores (both *P* < 0.01). We did not find significant difference in people with different marriage status (Table 2).

After adjustments for age and education, provinces in China showed different practice scores. Inner Mongolia, Xinjiang, Liaoning, Tianjin, and other northern provinces had higher practice scores (Figure 1C). There was no significantly difference in practice scores between Hubei Province and other provinces (β = −0.1, *P* = 0.07) (Table 2).

### Anxiety scores in people with different characteristics

We found that 90.3% (8,815/9,764) of participants had experienced varying levels of anxiety. Thirty-six percent (3,518/9,764) people reported that they were frequently angry, and 33.8% (3,304/9,764) people often had pessimism. Only 5.0% (486/9,764) of people were not nervous about the outbreak. Among all participants, the median anxiety score was 11 (P_25_ = 8; P_75_ = 15). The proportion of women (30.6%) in high anxiety state was higher than that of men (21.0%). With the increase of age, the anxiety of the population gradually decreased (β = −0.2, *P* < 0.01). With the increase of education, the proportion of people with high anxiety level rose gradually (15.3%, 20.7%, 28.3%, and 31.4% for junior high school or above, senior high school, bachelor’s degree, and master’s degree or above, respectively); (Table 2). In addition, medical professional (32.0%) and people living in urban area (28.1%) had higher proportion in high anxiety than other people.

Based on the standardized anxiety scores, we found people in Hubei Province were the most anxious (37.2% with high anxiety), followed by those living in Beijing (30.5% with high anxiety) and Shanghai (30.2% with high anxiety) (Figure 1D). The results of GLM showed that the anxiety of people in Hubei Province (mean, 12.6) was much higher than that of other provinces in China (mean, 11.5, *P* < 0.01) and abroad (mean = 11.7, *P* < 0.05) (Table 2).

### Perceived risks and behavior in wearing masks and hand hygiene

In this study, 86.5% of people in Hubei Province reported that they have been in contact with someone with a confirmed or suspected case, compared with only 6.5% of people in other provinces. Compared to females, males did not want to wear a mask to go out (4.2% vs 2.2%; OR 0.5; 95% CI, 0.4–0.6) or clean their hands frequently (5.4% vs 3.1%; OR 0.5; 95% CI, 0.4–0.7). People over 30 years were more likely to wear masks than those under 30 years (OR 2.4; 95% CI, 1.8–3.4 for 30–39 year olds, OR 2.7; 95% CI, 1.9–3.9 for those aged 40–49 years, and OR 2.2; 95% CI, 1.6–3.2 for those aged ≥50 years). The perceived risk and the possibility of wearing masks and hand hygiene of the people in Hubei Province were not significantly different from those in other provinces (*P* = 0.76, *P* = 0.66, and *P* = 0.32, respectively). After adjusting for gender, age, education, and province, people with risk behaviors could clearly know that they were at high risk (β = 0.3, *P* < 0.01). However, people who had experienced risk behaviors were less likely to wear masks and practice good hand hygiene than those without risk behaviors (OR 0.6, *P* = 0.03 and OR 0.8, *P* = 0.19, respectively) (Table 3).

**Table 3.** Ability to perceive risk and behavior in wearing masks in people with different characteristics especially those with risk behaviors, China, 2020

Characteristics	Number	Perceived risks			Wearing masks		Cleaning hands	

		Mean	β	*t*	*N* (%)	OR (95% *CI*)	*N* (%)	OR (95% *CI*)
**Gender**								
Female^Ref^	6,486				6,342 (97.8)		6,286 (96.9)	
Male	3,278	2.2	0.0	−1.7	3,140 (95.8)	0.5 (0.4–0.6)^b^	3,100 (94.6)	0.5 (0.4–0.7)^b^
**Age group, years**								
<30^Ref^	2,725				2,599 (95.4)		2,591 (95.1)	
30–39	2,858	2.2	0.1	2.4^a^	2,799 (97.9)	2.4 (1.8–3.4)^b^	2,772 (97.0)	1.8 (1.3–2.3)^b^
40–49	2,382	2.2	0.0	1.0	2,333 (97.9)	2.7 (1.9–3.9)^b^	2,314 (97.1)	2.2 (1.6–2.9)^b^
≥50	1,799	2.2	−0.1	−2.8^b^	1,751 (97.3)	2.2 (1.6–3.2)^b^	1,709 (95.0)	1.3 (1.0–1.7)
**Education**								
J&B^Ref^	483				441 (91.3)		415 (85.9)	
Senior high school	1,315	2.0	0.0	1.1	1,258 (95.7)	2.3 (1.5–3.6)^b^	1,227 (93.3)	2.5 (1.8–3.5)^b^
Bachelor’s degree	5,549	2.0	0.2	5.2^b^	5,422 (97.7)	5.1 (3.5–7.4)^b^	5,396 (97.2)	6.4 (4.7–8.8)^b^
Master’s degree or above	2,417	2.2	0.2	5.3^b^	2,361 (97.7)	5.2 (3.4–8.0)^b^	2,348 (97.1)	5.9 (4.1–8.5)^b^
**Area**								
Hubei province^Ref^	156				151 (96.8)		146 (93.6)	
Other provinces in China	9,408	2.5	0.0	−0.3	9,153 (97.3)	0.8 (0.3–2.1)	9,047 (96.2)	1.4 (0.7–3.0)
Abroad	200	2.2	−0.1	−1.4	178 (89.0)	0.2 (0.1–0.4)^b^	193 (96.5)	1.3 (0.5–3.8)
**Risk behaviors**								
No^Ref^	9,006				8,757 (97.2)		8,666 (96.2)	
Yes	758	2.1	0.3	10.0^b^	725 (95.6)	0.6 (0.4–1.0)^a^	720 (95.0)	0.8 (0.5–1.1)

CI, confidence interval; J&B, junior high school and below; OR, odds ratio; Ref, reference group.

^a^*P* < 0.05.

^b^*P* < 0.01.

### Interest and channel preference for epidemic information of respondents

**Figure 2.**
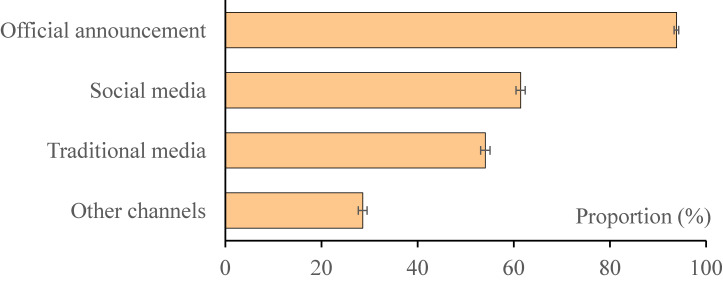
Channel preference for COVID-19 outbreak information reporting and seeking, China, 2020

Most (97.6%; 9,525/9,764) of the respondents paid attention to the epidemic information every day; 79.0% (7,717/9,764) believed the outbreak would be fully controlled within 3 months; and 93.9% (9,165/9,764) of the respondents obtained epidemic information through official announcement, followed by social media (61.4%) and traditional media (54.1%) (Figure 2).

## DISCUSSION

Our study demonstrated that this COVID-19 outbreak had attracted widespread public attention in China, with nearly 98% of the population continuously tracking the situation, and nearly 80% of them had the confidence that the government could control the outbreak. More than two-thirds of the public had high level awareness of COVID-19 and the precaution measures; however, regional differences exist. At the beginning of the COVID-19 outbreak in China, people in Hubei, Beijing, and Shanghai had more anxiety than those in other provinces.

At the beginning of the COVID-19 outbreak, a rapid response and an open, transparent system for epidemic reporting and news briefing are of great significance to improve people’s confidence in epidemic prevention and control. The epidemic can generally be controlled effectively through syndromic (fever) surveillance, contact tracing, quarantine, and self-protection, just like SARS-cov and MERS-cov.^19^ Therefore, China had strongly restricted movement across Hubei Province and then escalated it to the rest of the provinces in a short period.^20^ Our research found that the internet media and mobile media played a very important role in the publicity and communication of epidemic prevention and control, with more than two-thirds of the respondents getting the outbreak news and official announcements through online media, such as official website, Weibo, and WeChat. In China, there were 112.2 mobile phones per 100 people on average in 2018,^21^ which provides a guarantee for the government to guide people to strengthen self-protection online. In fact, more than three-fourths of people accurately understood the transmission route, incubation period, and preventive measures of COVID-19 because of the spread of official information. It is very important for people to know the accurate information of the disease, especially the prevention measures, which play an important role in the prevention and control of the outbreak. In the study, 95.4% of the respondents knew the incubation period of COVID-19. In contrast to China, a study from Saudi Arabia showed that people had a high awareness of transmission routes and clinical symptoms of MERS-CoV but a low awareness of its incubation period.^22^ Young adults appeared as being more active in terms of knowledge. We considered that this difference may be caused by the popularity of mobile phones and the internet. About 50% of people currently infected with COVID-19 are over 50 years old in China.^23^ Therefore, what deserves our attention is the improvement of epidemic knowledge of people over 50 years old, especially in Hubei Province.

We found that 66.9% of respondents were able to wear appropriate masks to prevent COVID-19 infection, 81.5% were able to change their masks regularly, and 96.1% of people maintained hand hygiene and washed their hands frequently. WHO and United States Centers for Disease Control and Prevention does not recommend wearing masks to prevent infection and suggests hand-washing as a protective measure for general population as of the completion of this investigation.^24^^,^^25^ However, based on Chinese guidelines, we could conclude that more than 30% of respondents were not able to wear appropriate masks to prevent COVID-19 infection, and nearly 20% did not change their masks regularly. The proportion of people taking preventive measures is similar to that among the population in Reunion Island during the influenza A (H1N1) pandemic,^26^ but still low. Previous literature showed that the relationship between age and preventive behavior may change with different diseases and different population.^26^^–^^28^ Reducing the misunderstanding of personal protection is the focus of public awareness and health education in the next step.

Our study confirmed that anxiety related to COVID-19 outbreak was common in the general population. Anxiety and fear has a negative impact not only on health, but also has economic effects and social consequences, as was seen during the SARS outbreak in 2003.^29^ In addition to Hubei Province, people in multiple provinces, like Shanghai and Beijing, also had experienced much anxiety. Cities that experienced previous outbreaks might be more sensitive to the epidemic of infectious diseases.^30^^,^^31^ Younger people living in urban areas or with higher education were more anxious about the outbreak, which showed similar trends observed for anxiety and panic during the H5N1 avian influenza pandemic in Hong Kong^32^ and during the SARS outbreak in Qatar.^33^ While COVID-19 is still spreading across the country, the public generally experiences high levels of panic, fear, anxiety, and irritability. These negative emotions can progress into behavioral changes, such as being afraid to leave the house, blindly disinfecting, and scrambling for medicines. Baidu, one of the most common search engines in China, has released an information index, for which “psychological counseling” in Hubei Province increased by 3,275% from January 20th to January 26th, and the search index increased by 51% in the 7 days.^34^ In this study, 86.5% of people in Hubei Province reported that they had contacted high-risk population or environment, compared with only 6.5% of people in other provinces. Besides, many areas have cut off traffic with Hubei. This would increase their psychological stress. These results suggested we should not only support Hubei Province in medical treatment, but also need to strengthen health education and psychological counseling. It is recognized that extensively implementing mental health monitoring in the community is a worthwhile strategy. Community workers should regularly assess the psychological status of community families and report to their superiors. Meanwhile, the government should strengthen media efforts to ensure the validity and accuracy of output information. We strongly recommend that the government develop health education strategies to address mental health issues, promote healthy behaviors, and reduce psychological stress.

In this study, people with risk behaviors clearly know that they were at high risk, but they did not pay enough attention to wearing a mask when going out and maintaining good hand hygiene. According to local regulations, people must wear masks when going out. What’s more, maintaining hand hygiene is an effective protection for themselves. We consider that appropriate risk perception of the outbreak can lead to beneficial changes in health behaviors, such as wearing masks and washing hands frequently. And strong publicity and punishment may increase the possibility of wearing masks and other protective behaviors. However, due to the sudden outbreak, the supply of masks in China is insufficient to meet the needs of some people.

There are some limitations to our study. First, online questionnaires led to a biased selection. The respondents are mainly those living in urban area and with high school education or above, which may overestimate the knowledge of the outbreak and protection. Second, although we have carried out quality control, there may be errors in the information because the online questionnaire cannot be modified after filling in. Third, this study is a cross-sectional survey and cannot show trends. In order to dynamically assess knowledge, practice, and psychological pressure of Chinese people on the outbreak, we have conducted a follow-up survey while the epidemic was under control.

In summary, the public was very concerned about the COVID-19 outbreak, with high knowledge of the transmission route and incubation period of the disease, and a high proportion of people practiced good hand hygiene behavior; however, quite a number of people experienced anxiety caused by quarantine or mobility control, especially in Hubei Province. The findings suggest the importance of closing the gap between knowledge and good practice, and the need to reduce anxiety caused by the pandemic. Conducting psychological counseling and health education to public and patients is an important and key measures to address the public anxiety issue.

## APPENDIX A. SUPPLEMENTARY DATA

eFigure 1. Timeline of COVID-19 events

eFigure 2. Inclusion and selection process of respondents

eMaterial 1. Questionnaire on awareness, prevention practice and psychological effect of COVID-19 in 2020
