# Supplementary material for: Public Awareness, Individual Prevention Practice, and Psychological Effect at the Beginning of the COVID-19 Outbreak in China
Source: J Epidemiol. 2020 Oct 5;30(10):474–82. doi: 10.2188/jea.JE20200148 (PMC7492706; doi:10.2188/jea.JE20200148)

eFigure 1. Timeline of COVID-19 events

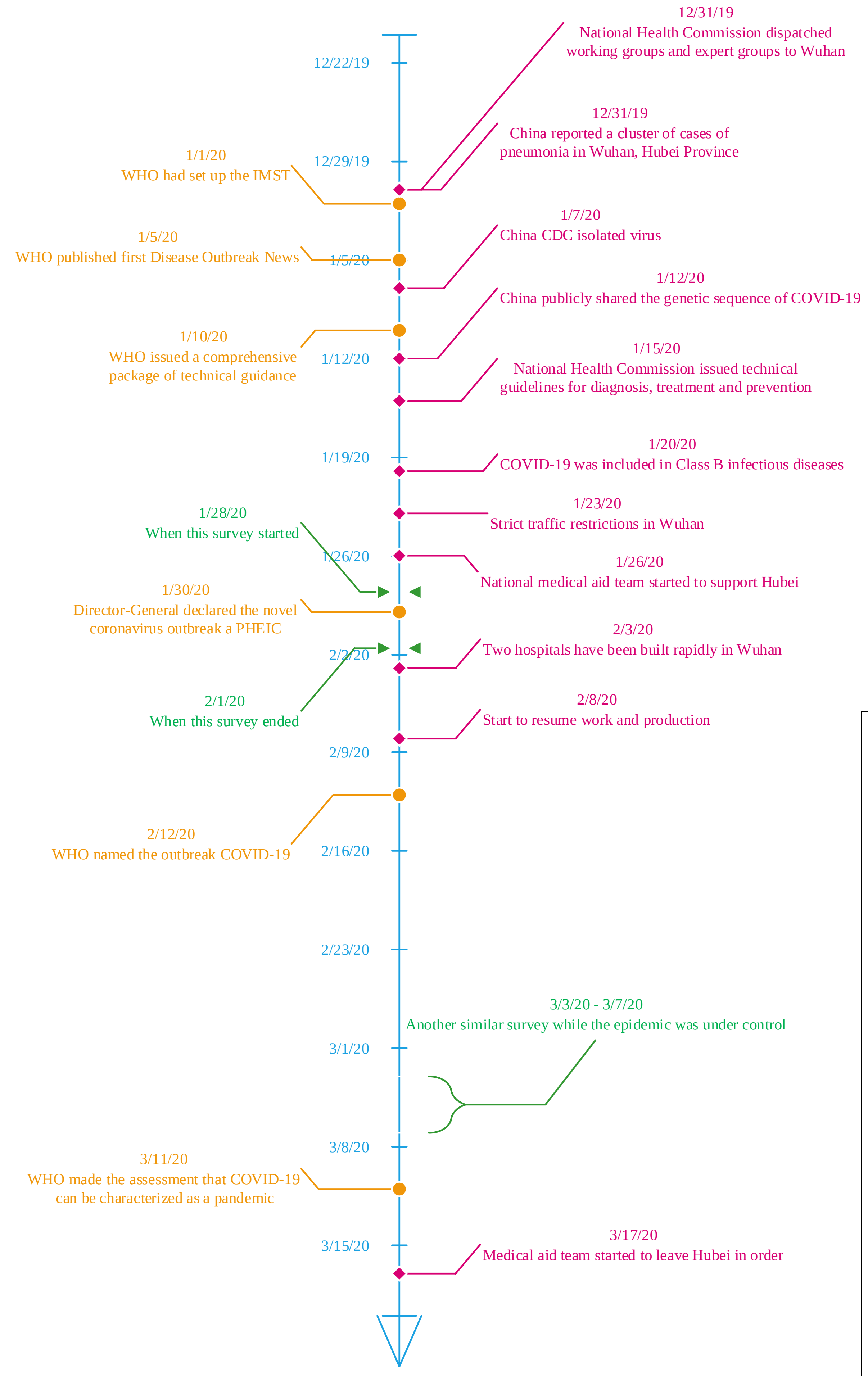

References:
[1] WHO Timeline - COVID-19 [homepage on the Internet]; [updated 2020-04-27]. Available from: https://www.who.int/news-room/detail/27-04-2020-who-timeline---covid-19
[2] Record of COVID-19 epidemic information in China [homepage on the Internet]; [updated 2020-04-06]. Available from: http://www.gov.cn/xinwen/2020-04/06/content_5499625.htm

eMaterial 1. Questionnaire on awareness, prevention practice and psychological effect of COVID-19 in 2020

Informed Consent

There is an online survey, designed by School of public health, Peking University, aiming to understand the public knowledge, practice of prevention, and psychological status of Corona Virus Disease 2019(COVID-19). This survey does not involve any privacy or sensitive issues, and the results of the it will be helpful to China's epidemic management.

Part A. Relevant information acquisition and personal risk perceptions of COVID-19

A1. How often are you concerned about the outbreak?

a. Every day

b. Occasionally

c. Don't care

A2. Where do you get information about the outbreak?

[You can choose more than one]

a. Government and professional organizations (including official websites, Weibo and WeChat)

b. News media

c. Search engines (Baidu, Google, etc.)

d. Personal social media

e. TV

f. Radio

g. Community brochure/brochure/column, etc

h. Other mass media (newspapers/magazines)

i. Other (please indicate)________

A3. Do you trust the unofficial release of information online? (unofficial: like the grapevine from friends)

a. Completely trust

b. Some trust

c. General trust

d. Some distrust

e. Thoroughly distrustful

A4. How long do you think it will take to control the outbreak with the current prevention and control efforts?

a. Within a month

b. 2-3 months

c. 4-6 months

d. Over 6 months

e. I don't know

A5. Were you exposed to the following situation recently?

[You can choose more than one]

a. Visited Hubei/Wuhan

b. Contacted with Hubei/Wuhan people

c. Lived in the environment with confirmed or suspected cases

d. Visited the wildlife trade market

e. No above

A6. What do you think is your personal risk of infection with COVID-19?

a. No risk

b. Low risk

c. Medium risk

d. High risk

e. Extremely high risk

A7. How did you feel nervous or anxious in the latest week?

a. Frequently

b. Often

c. Generally

d. Less

e. Hardly ever

A8. How did you worry about getting sick in the latest week?

a. Frequently

b. Often

c. Generally

d. Less

e. Hardly ever

A9. How were you prone to anger in the latest week?

a. Frequently

b. Often

c. Generally

d. Less

e. Hardly ever

A10. How were you pessimistic in the latest week?

a. Frequently

b. Often

c. Generally

d. Less

e. Hardly ever

A11. How did you feel tired in the latest week?

a. Frequently

b. Often

c. Generally

d. Less

e. Hardly ever

Part B. Knowledge of COVID-19

B1. By which way may the novel coronavirus 2019(2019-nCoV) infect humans?

[You can choose more than one]

a. Client sneezes, coughs, and speaks in droplets, exhaled gas inhaled at close range

b. Droplets mix in the air to form aerosols that are inhaled by susceptible people

c. Droplets deposited on the surface of the object, contact with the pollution of the hand, then contact with the mouth, nose, eyes and other mucous membrane

B2. How many days of medical observation are required for close contacts of COVID-19 infection?

a. 7 days

b. 10 days

c. 14 days

d. 21 days

B3. Are asymptomatic patients with COVID-19 infectious?

a. Yes, they are contagious

b. No, they are zero infectivity

c. I don't know

B4. Who is susceptible to COVID-19?

[You can choose more than one]

a. The elderly

b. The middle-aged

c. Young adults

d. Children

e. I don't know

B5. What kind of situation should people be vigilant?

[You can choose more than one]

a. With a history of exposure to patients with COVID-19

b. With fever, fatigue, dry cough, progressive difficulty breathing

c. With headache, dizziness

d. With abdominal pain, diarrhea and other gastrointestinal symptoms

e. With travel history from affected areas

B6. What would you do if you developed symptoms related to COVID-19?

a. Home isolation, self-medication

b. Go to the nearest clinic

c. To open the medical treatment of fever clinic

d. I don't know

Part C. Individual Protection

C1. What kind of mask do you think can effectively prevent 2019-nCoV infection?

[You can choose more than one]

a. Surgical mask

b. Medical mask (N95 and above)

c. Gauze mask

e. Activated charcoal mask

f. None of them/I don't know

C2. In which environment would you wear a mask?

[You can choose more than one]

a. Parks and roads

b. Supermarkets, malls and other crowded places

c. Small confined Spaces such as elevators

e. Hospital

f. None of them/I don't know

C3. Is there a time limit for the effectiveness of mask wearing?

a. No

b. Yes, 2-4 hours

c. 5-6 hours

d. I don't know

C4. Can hand washing prevent 2019-nCoV infection?

a. Yes

b. No

c. I don't know

C5. What measures have you taken to protect yourself from 2019-nCoV?

[You can choose more than one]

a. Wear a mask when going out

c. Do not use hands when sneezing or coughing

d. Keep hands clean by washing hands correctly and timely

e. Do not go to parties or visits, and try to avoid crowded or enclosed places

f. Eat a healthy balanced diet

Part D. Sociodemographic Characteristics

D1. Are you currently living in an urban or rural area?

a. Urban

b. Rural

D2. What is your age?

a. [number]

D3. What was your sex at birth?

a. Male

b. Female

D4. What is your marital status?

a. Never married

b. Engaged or married

c. Separated or divorced

d. Widowed

e. Other

D5. What is your highest level of education?

a. Primary school

b. Junior high school

c. Senior school

d. Some college or a bachelors degree

e. Masters, PhD, or above

D6. What is your main occupation?

a. Labor worker (blue collar)

b. Farmer

c. Teachers and researchers

d. Service/retail

e. Civil servant

f. Health care workers

g. Individual operator

h. Office worker (white collar)

i. Driver

j. Retired people

k. Student

l. Unemployed

m. Other

D7. How many people are there in your family?

a. 1

b. 2

c. 3

d. 4

e. 5

f. 6

g. 7

h. 8

i. 9

j. 10

D8. Are there any children (under 5) in your family?

a. Yes

b. No

D9. Are there any elderly men or women (over 65) in your family?

a. Yes

b. No

D10. Are there any pregnant women in your family?

a. Yes

b. No

D11. How is your general health?

a. Good

b. About good

c. Fair

e. Bad (under illness)

eFigure 2. Inclusion and selection process of respondents

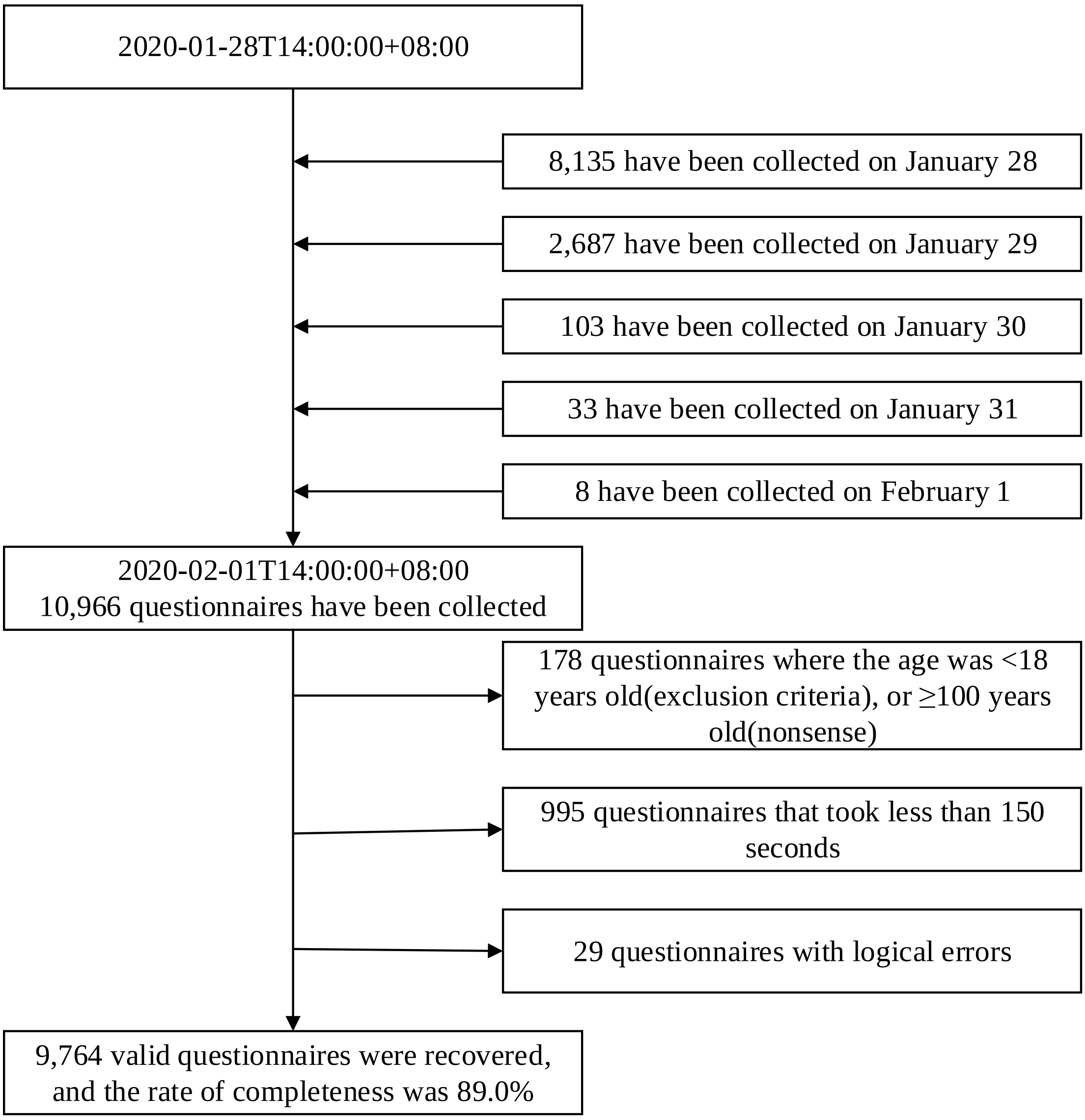

Supplement: Supplementary file 1 [file je-30-474-s001.pdf]
